# Assessing the motivation to learn in cattle

**DOI:** 10.1038/s41598-020-63848-1

**Published:** 2020-04-22

**Authors:** Rebecca K. Meagher, Emma Strazhnik, Marina A. G. von Keyserlingk, Daniel M. Weary

**Affiliations:** 1 Animal Welfare Program, University of British Columbia 2357 Main Mall, V6T 1Z4 Vancouver, Canada; 2 Present Address: Department of Animal Science and Aquaculture, Dalhousie University 58 Sipu Road, B2N 5E3 Bible Hill, N.S. Canada

**Keywords:** Animal behaviour, Psychology

## Abstract

Cognitive challenges may provide a form of enrichment to improve the welfare of captive animals. Primates, dolphins, and goats will voluntarily participate in learning tasks suggesting that these are rewarding, but little work has been conducted on livestock species. We investigated the motivation of 10 pairs of Holstein heifers to experience learning opportunities using a yoked design. All heifers were trained to perform an operant response (nose touch) on a variable interval schedule. Learning heifers then performed this response to access a discrimination learning task in which colour and texture of feed-bin lids signified a preferred reward (grain) vs. a non-preferred reward (straw). Control heifers received the same feed without a choice of bins or association of feed with lid type. Learning heifers approached the target to begin sessions faster (p = 0.024) and tended to perform more operant responses (p = 0.08), indicating stronger motivation. Treatments did not differ in the frequency with which heifers participated in voluntary training sessions. We conclude that heifers are motivated to participate in learning tasks, but that aspects of the experience other than discrimination learning were also rewarding. Cognitive challenges and other opportunities to exert control over the environment may improve animal welfare.

## Introduction

Learning has an obvious adaptive function. Animals must learn how and where to obtain food and other biologically important resources in the short-term; they can also acquire information about their environments that might aid in survival or acquisition of resources in the future through exploration or social learning (see e.g.^1^). This information-gathering function is assumed to explain the motivation to explore exhibited by many species (e.g.^2,3^). Some researchers have suggested that learning itself might be rewarding for animals, and opportunities to use cognitive abilities might be important for the welfare of captive animals^4^.

There is evidence that animals will voluntarily engage with learning tasks. For example, goats continued to engage with a computerised discrimination learning task to earn water even when water was freely available elsewhere; those that were most successful at learning preferentially used the learning device rather than drinking from the free water source^5^. This result is consistent with older work on primates that reported that monkeys would sometimes interact with puzzles even when no external reward was offered or discard food rewards to continue working on the puzzle (reviewed by^4^). Some more recent research on cetacean and primate cognition relies on individuals voluntarily participating in training sessions (e.g.^6,7^).

Compared to primates and cetaceans, less attention has been paid to farm animals like cattle that are often viewed as less intelligent (reviewed by^8^). However, cattle have the ability to learn about their social companions and features of their environments such as patterns of food availability^9^. The results of a study by Hagen and Broom^10^ suggested that the experience of learning might matter to cattle; these authors trained Holstein-Friesian heifers to press a panel to access a runway with a feed reward at the end. Heifers had an increased heart rate during sessions in which their learning performance improved, and tended to move more vigorously as compared to control heifers who were allowed into the runway without having to perform any specific behaviour. Hagen and Broom^10^ cautiously interpreted this finding as evidence of a positive emotional response (‘excitement’). They also noted that some heifers who had learned the operant response exhibited play behaviour after the gate opened, a finding only observed once in a control heifer. A similar “Eureka effect” was described in dogs by McGowan and colleagues^11^, in which dogs wagged their tails more often in the learning condition than in the control condition where the same food was received but delivery was unpredictable.

Our aim was to test the hypothesis that cattle value opportunities to learn. We predicted that cattle would be more motivated to learn how to obtain a preferred feed than to simply have that feed presented to them, as indicated by performance of operant responses, shorter latencies to engage in training, and increased rates of voluntary participation in the training. We also predicted that play would increase in association with successful learning, and that motivation to engage in the learning task would increase as animals became more successful.

## Results

### Dropouts

Of the initial 30 heifers, 5 were dropped because they did not meet the participation criterion of receiving grain with a click 20 times and eating it at least 5 times during clicker training (i.e. they never engaged with the training) after 25 sessions. An additional 3 were dropped because they stopped engaging, as indicated by at least 10 sessions in which they did not eat in a given phase of training: one during learning of the operant and two during the adjustment to entering the training arena. One heifer was dropped because her partner stopped engaging, and one was dropped during the discrimination phase because training could not be completed by the end of the study, partly due to poor engagement. In total, 20 heifers completed the study.

### Relationships among dependent variables

Within the discrimination learning phase there was a negative rank correlation between latency to approach the lid and average number of operant responses per session during the discrimination phase; i.e. individuals slowest to approach the area where they could initiate interactions with the operant target also performed the fewest responses over the course of the session (r_s_ = 0.63; p = 0.003). Neither latency nor operant responses per session correlated with the proportion of offered voluntary sessions in which the heifer chose to participate (latency: r_s_ = 0.29; operant: r_s_ = 0.13; both p > 0.10).

### Treatment effects on motivation

**Figure 1:**
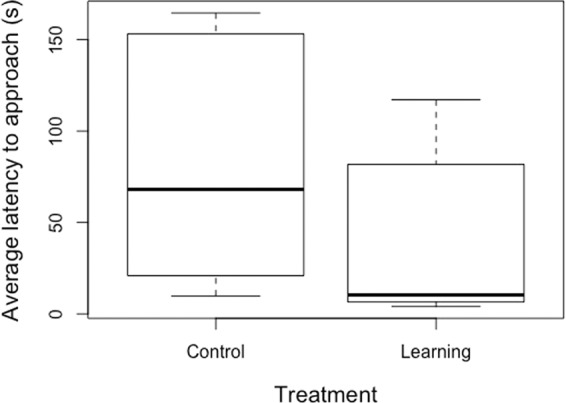
Box plots of average latency for heifers to approach the training area during the discrimination learning phase, shown separately for the Control and Learning treatments (n = 10 per treatment).

**Figure 2:**
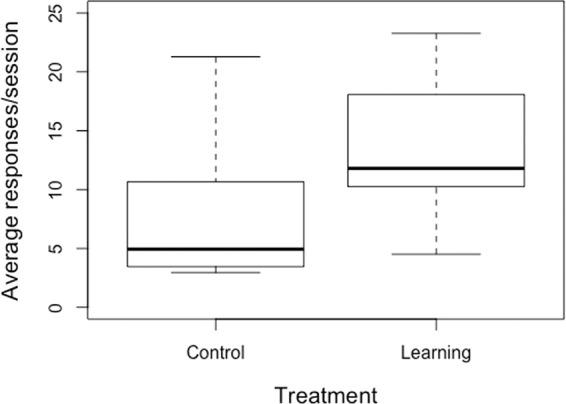
Box plots of average number of operant responses per session, shown separately for heifers in the Control and Learning treatments (n = 10 per treatment).

The latency to approach the operant target during the discrimination learning stage was shorter for Learning than Control heifers (back-transformed least squares means 20.7 vs. 56.8 s; t = −2.4, d.f.=16, p = 0.02; Fig. 1). This remained true after excluding the pair that had been treated for illnesses (t = −2.2, denominator d.f.=14, p = 0.04). The number of operant responses performed per session tended to be higher in the Learning treatment (least squares means 12.1 vs. 7.6; t = 1.9, denominator d.f. = 16, p = 0.08; Fig. 2); this tendency disappeared if the sick pair was excluded (p = 0.16). All heifers participated in at least one voluntary session during the experiment, and just one of the 20 did not do so during the discrimination learning stage. Across both treatments, participation in this stage ranged from 0 to 100% of offered sessions with a median of 63.3%; this percentage did not differ between treatments (logistic regression denominator d.f. = 15, p = 0.29; odds of participation as ratio of Learning:Control = 1.6 [95% CI: 0.7–3.8]).

Latency to approach did not differ between sessions that contributed to meeting the learning criterion and sessions before the criterion was met (median 9.6 vs. 12.1 s; Wilcoxon signed rank V = 27, p = 1). Mean number of operant responses performed per session was higher during the sessions that contributed to the learning criterion than the preceding sessions (mean of the differences: 2.8 [95% CI 0.01–5.6]; paired t-test, t = 2.3, p = 0.05).

### Play

During the baseline period, there was no difference between treatments in the occurrence of play, with the median for the Learning group being 1.0 occurrences per session (IQR: 0.6–1.6) vs. 1.0 (0.1–2.1) for the Control (p = 1.0). There was also no difference during discrimination learning (median [IQR] Learning: 2.7 [1.1–5.9] vs. Control 3.0 [2.3–4.0], p = 1.0). Expression of play behaviour was highly variable within and among heifers during the discrimination stage (see Supplemental Fig. 1 for comparison with learning performance).

## Discussion and Conclusions

We found some evidence that heifers are motivated to participate in training sessions. Almost all heifers who completed the experiment voluntarily ‘participated’ (i.e. chose to enter the training alley), indicating that some aspect of the experience was rewarding. Latency to approach the operant target and number of operant responses could be considered the most direct evidence of motivation to participate in the discrimination learning task, and both of these measures showed differences in the predicted direction (although only latency was significant). The lack of a significant difference for latency may be because the overall experience was positive even for Control heifers. Our other proposed indicator of motivation, voluntary participation in sessions, was assessed before or at the time of leaving the home pen, and thus may have been influenced by motivation to enter the alley for other reasons such as having more space for locomotion and exploration. Learning to perform the operant response itself might have been rewarding. The fact that voluntary participation did not correlate with latency or number of operant responses performed, while those two variables were correlated with one another, supports the interpretation that voluntary participation reflects a different motivation.

Relatively little empirical work has been done on the welfare benefits of cognitive enrichment. Voluntary participation suggests that engaging in learning tasks can be rewarding, but it is difficult to say how often or for how long animals need to engage for this to be considered beneficial. What little data has been collected on other welfare indicators has been somewhat equivocal. For example, Clark and Smith^12^ reported more social play among chimpanzees when a puzzle device was present, and Whitehouse and colleagues^13^ similarly found some improvements in social interactions among macaques on training days compared to non-training days. Zebunke and colleagues^14^ found some evidence that pigs exposed to an acoustic discrimination task were less fearful and more exploratory. A recent study in dolphins found willingness to participate was positively correlated with health status and negatively with indicators of likely agonistic interactions with other dolphins^15^, but willingness to participate was posited to decline as a result of pre-pathological states rather than being a causal factor in welfare. The heifer work completed over a decade ago^10^ was suggestive, but required supporting evidence given that there were limited data for play, and changes in arousal alone cannot be assumed to reflect a positive affective state. The current study addressed these limitations by contributing data on play, and assessing motivation rather than physiological arousal. It is typically argued that welfare is likely to be improved by providing animals with things they are motivated to have (see^16,17^), so the data presented here are consistent with the idea that welfare is improved by this training opportunity.

There was no treatment effect on play in the current study, and unlike Hagen and Broom^10^, we observed play in both learning and control individuals. Play bouts were rare but did increase slightly in frequency over time, as reflected in the higher levels reported during the discrimination learning stage. This increase may have reflected reduced fear of the training environment or some loss of interest leading to off-task behaviour. Play behaviour was least frequent in the two heifers who learned the task fastest, perhaps because they were more focused. Due to the difficulty in identifying the moment at which heifers learned the association, it is impossible to associate differences in play with a clear ‘eureka’ moment. Our results provide no evidence that play is linked with a learning experience. Future work could look at play over the full day as an indicator of more lasting effects on affective states; during the task when most animals were engaged in other activity, rates were not likely reflective of overall welfare impact of the treatment.

This study brings together different lines of evidence that animals benefit from exposure to a cognitive challenge. The results suggest that voluntary participation may not always be directly tied to motivation to experience the cognitive challenge; the relevance of voluntary participation will likely depend on experimental design, with participation being more straightforward to interpret in designs involving learning devices freely available in the home pen. Regardless, when participation comes at no cost, it cannot be used to draw inferences regarding the strength of the motivation. One other advantage of including some cost to engaging in the task is that it helps to separate two features of learning that might both contribute to welfare; cognitive engagement and increased control over the environment are typically provided by successful learning, and both are expected to have benefits for animal welfare^4,5^. Exerting control is a form of agency, which is suggested to be important for good welfare even beyond the affective consequences of achieving desired outcomes^18^. Where animals given the opportunity to learn are compared to controls who do not have to perform any response in order to receive the reward (e.g.^11^), only the learning treatment are able to exert this type of control. In contrast, in the present experiment, all heifers learned the initial operant response, and were in this way exerting control over their environment. In voluntary sessions, they were also exerting control by choosing if and when to enter the arena. This control is one potential explanation for the willingness of heifers to participate regardless of treatment, as has been suggested for cattle’s willingness to perform physical work for freely available feed (i.e. contrafreeloading^19^). The difference in motivation between treatments, here indicated by the different latency to approach, could be attributed to Learning heifers being able to learn something more complex and thus may having greater continued cognitive engagement during the discrimination learning stage. An alternative interpretation of this difference relates to predictability; animals typically prefer signalled to unsignalled rewards^20,21^. When heifers successfully learned the discrimination, the reward they received would be predictable, whereas Control heifers were not able to predict which reward they would receive. Future experiments could distinguish between effects of cognitive challenge and predictability by continued testing: if cognitive stimulation is key then peak motivation should occur around the time of learning and afterwards decline. In contrast, if predictability is key then treatment differences should persist even after a long period of testing.

It is worth noting that nine of 30 heifers (30%) were excluded for failing to participate at some stage (while a tenth heifer was dropped due to her partner’s failure to participate). For those heifers excluded at early stages of the training (e.g. clicker training), the problem typically appeared to be fear rather than lack of interest; qualitatively, heifers were judged to be in vigilant states, appeared hesitant to approach the training area or were unwilling to leave the home pen. For those who ceased to respond at later stages, the underlying motivation was more difficult to judge; these animals may have had a reduced interest in the task or may have experienced frustration with no longer being rewarded immediately on every occasion for performing the operant. Previous discussions of cognitive enrichment have highlighted that the benefits of cognitive challenge depend on the ability to meet that challenge; a task that is too difficult could cause anxiety or frustration, and it is therefore recommended that appropriateness of the context be carefully considered and welfare monitored at the individual level^4^. The current study makes it clear that complex tasks might not be appropriate for all individuals, so animals should not be forced to participate in training for the purposes of “cognitive enrichment”. This might be an even more common problem in systems where cattle are individually reared, unlike those in this study, since our previous work indicates that individual housing can impair performance in complex learning^22,23^. However, if reluctance to participate is due to fear rather than task difficulty, it may be possible to introduce such enrichment gradually and avoid fear responses.

To conclude, offering learning opportunities may provide welfare benefits to most heifers, but it is unclear if the complexity of the learning or other aspects of the training experience (including increased control) are most important. We found no evidence of a clear ‘eureka!’ moment, and no clear association between learning and play.

## Methods

### Subjects and housing

Thirty Holstein heifers were enrolled in the study, ranging in age from 5 to 10 months, divided into two cohorts. These heifers were reared indoors in group pens. During the trial, heifers were housed in deep sand-bedded free stall pens (12.1 × 6.7 m) at the UBC Dairy Education and Research Centre, at a stocking rate of less than one heifer per stall, and fed a total mixed ration consisting of 42.36% rye grass silage, 37.66% alfalfa hay, and 19.98% concentrate (Hi-Pro Feeds grain mixture), provided twice daily at approximately 6:00 and 15:00 h. Water was provided via a self-filling trough. This study was performed in accordance with all relevant Canadian legislative and regulatory requirements. Animal care provided met the standards of the Canadian Code of Practice for Dairy Cattle. All experimental procedures were approved by the UBC Animal Care Committee (Protocol A120337).

### Treatments

All heifers were trained to perform an operant response (a nose touch) to gain access to a testing arena. Using a yoked design, half of the heifers (the ‘Learning’ group) were then given an opportunity to learn a discrimination task while in this arena; ‘Control’ heifers received the same feed rewards as their yoked partners in the learning group, but without the opportunity to learn how to obtain the preferred feed. Heifers were assigned to treatments alternately within each pen based on the order in which they reached the step of training in which discrimination learning could begin. If two heifers from a pen reached this step on the same day, the assignment was randomized.

**Figure 3:**
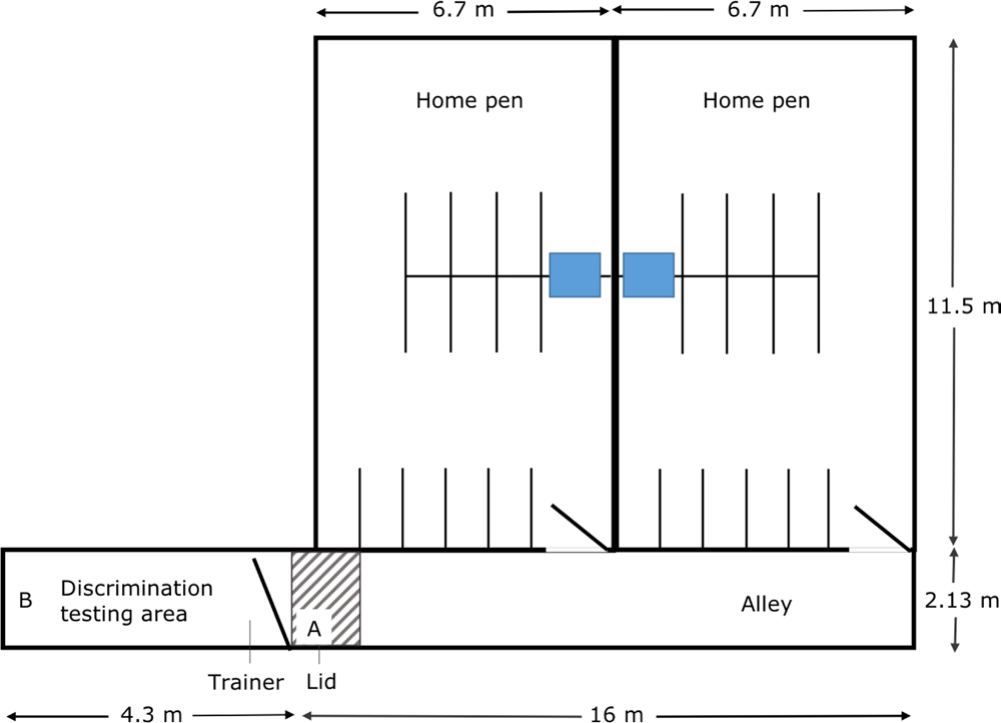
Layout of pen and area used for learning experiment. The feed bin was placed in location A during initial training and location B by the discrimination learning stage. The lid was the operant target, suspended at eye level. The area marked by diagonal stripes is the “training area”.

Testing was conducted in the alley behind the home pen (Fig. 3). At the end of the alley was a plywood gate with a small (23 × 28 cm) window. The primary observer (‘trainer’) remained behind this gate, which opened to the discrimination learning arena. The plywood barrier at the far end of this arena was 40 cm above the floor, allowing the second researcher to place and remove the bins without entering the arena.

### Training

Training the heifers in preparation for the final discrimination task involved four main steps: habituation, clicker training for operant conditioning, operant response and adjustment to a variable interval schedule, and entry into the discrimination arena (for additional details on training methods, please see ESM). Habituation was completed by allowing the heifers into the training alley in a social group for two consecutive days. Daily training sessions then began. A feed bin was placed in the training area (see Fig. 1) and latencies to approach this area were recorded, with approach defined as the heifer’s head passing a post at the side of the alley approximately 1 m from the gate while facing in this direction.

Clicker training was conducted with heifers in pairs. When a heifer approached the feed bin, the trainer used a clicker to signal that a reward (a small handful of Hi-Pro Feeds grain mixture, consisting predominantly of barley, screenings grain and pellets with molasses and other additives) would be delivered. After the heifer had experienced at least 20 pairings of a click with grain, she moved on to learning the operant response.

From this stage onwards a transparent blue plastic lid (8.25 × 15.5 cm), suspended from a rope at the eye level of the heifer, was presented as the target. Heifers were rewarded for touching the rope or lid with their muzzles, using a click followed by a grain reward up to 10 times per session. Once this response was established, a variable interval (VI) schedule was introduced, such that heifers were rewarded for the first touch of the lid, after which there was an interval during which no rewards were available. The introduction of the variable intervals, which were randomly generated and ranged from 20–40 seconds with an average of 30 s (VI-30), allowed for differences in the number of responses performed to be assessed. Once heifers were proficient in the VI-30 schedule, heifers were trained to move through the open gate into the arena where discrimination testing would take place to receive the reward; this was done by progressively moving the feed bin toward this location over several sessions.

From the time the operant response was introduced, all heifers were given a maximum of six minutes to approach the training area when let into the alley. If they failed to enter the training area, they would be returned to the home pen and assigned a maximum latency of 361 s for the session.

#### Discrimination learning

When heifers had completed this training, they were assigned to the Learning or Control groups as described above and the discriminative stimuli were introduced. These stimuli were lids that heifers had to push off the feed bin, made of either plain white plastic or covered with a sheet of red foam. They thus varied both in colour and texture. In the case of the Learning treatment heifers, the red foam lid always signalled that the bin contained grain and would therefore be the ‘correct choice’, while plain white lids were placed on bins containing straw (a less preferred reward).

Heifers were first given a set sequence of “forced choice” trials in which a single feed bin was presented in the testing area, so that they were exposed to each type of lid and each feed. For Control heifers, the lid type was not associated with the feed it contained. For both groups bin placement varied to avoid any confound with location.

For the discrimination task, Learning heifers were then presented with two bins, one containing grain under the red foam lid and the other containing straw under the white plastic lid. The sides on which bins were placed varied in a pseudo-random pattern. The heifer was considered to have chosen when she moved a lid such that she could see the contents of the bin. Heifers were given five trials per session. The proportion of correct choices was calculated for each session; if learning heifers stopped participating before five trials were complete, sessions were pooled, and the proportion correct was calculated for each of the last three sets of five responses, regardless of breaks between sessions. Once a Learning heifer had completed three sets of five choices at 80% or more correct, she was given a probe session in which the bins were left empty until the choice was made to ensure she was not using scent or other cues from the reward. After a successful probe session (≥80% correct responses during the session), she was considered to have learned the discrimination task.

Control heifers were given only one bin containing one feed according to the sequence of feeds that her yoked learning partner chose in the corresponding session within this stage. The sequence of lid types was randomized with each lid type occurring at least once during each session so that the contents of the bin were not associated with the lid. Probe sessions were likewise given to match the Learning partner, with feed following the sequence the partner had received but not being placed in the bin until after the heifer had pushed the lid off.

### Outcome measures

Measures of heifers’ motivation to participate were: latency to approach the operant target when let into the alley to begin a session, number of operant responses performed per session, and how often heifers voluntarily participated in training sessions. Voluntary participation was assessed in extra training sessions held twice per week, beginning when the operant response had been established. The gate to the home pen was opened, and heifers were allowed to enter the alley voluntarily. Once one heifer had entered, the home gate was closed until her session was complete or she ceased to participate. When no animal entered the training alley within 5 min of the gate opening, voluntary sessions would cease for the day. The proportion of these sessions in which an individual chose to enter the alley was calculated. Since these measures of motivation have not previously been used in this species, convergent validity was assessed using co-variation among the measures. One aim of this study was to determine whether voluntary participation was a suitable indicator of motivation for the learning opportunity, since there is little information about how this relates to other measures of motivation. It was used as a simple test that can be compared across studies. Operant responses on a variable interval schedule are a more established measure of motivation in animal research (e.g.^24,25^), as is latency (e.g. latency to eat decreases with increasing hunger^26^).

Play behaviour during training sessions was also recorded to test for treatment effects and for changes among sessions in relation to learning success (based on Hagen and Broom^10^). Training sessions were recorded using overhead cameras. Videos were scored by the second author using continuous observation for each occurrence of any of the play behaviours described in Table 1.

**Table 1:** Play ethogram (adapted from^10,28^).

Jump	The front hooves are lifted off the ground, and hind hooves leave the ground at the same time. All four hooves may be lifted off the ground simultaneously. Top line descends from front to back.
Buck	Head is lower than shoulders. Hind hooves are lifted simultaneously, legs bent at the hock. The top line descends at a sharp angle from back to front.
Kick	As ‘buck’, with one leg bent at the hock and one leg extended sideways and back.
Rear	Front hooves lifted off the ground simultaneously, hind hooves remain on the ground. No locomotion is involved. Top line descends from front to back.

Heifers were observed on each training day for signs of respiratory or enteric illness. When illness was suspected, animals were referred to farm staff and treated according to the farm protocols as appropriate. One Control heifer was treated for coccidiosis during the discrimination learning period and the Learning heifer from that pair was treated for respiratory illness after completing learning. Analyses of treatment differences in motivation were repeated with this pair excluded to ensure that they did not influence the results.

### Analysis

Descriptive statistics and correlations between dependent variables were calculated using Minitab 17.3 (Minitab Inc. 2016). All other statistical analysis was done using R (The R Foundation for Statistical Computing, version 3.3.2, 2016) in RStudio (RStudio Inc., version 1.0.136, 2016). Sample code for the models used is presented in the Supplementary Material.

Relationships between measures of motivation during the discrimination learning phase were assessed using Spearman rank correlations due to non-normality. For each measure, we calculated average values for each individual across sessions. One average was calculated for a baseline period before the treatments differed, consisting of the sessions using the VI reinforcement schedule before introduction of the discriminative stimuli (lids); the second average was for the discrimination learning phase. To test whether the learning opportunity affected indicators of motivation, we then ran general linear models for each measure of motivation with the averages for the discrimination phase as the outcome variable, and the baseline value as a covariate to control for any pre-existing differences between individuals, and cohort (1 or 2) and treatment as predictors. For latency to approach, y-values were log-transformed to approximate normality. Proportion of voluntary sessions in which heifers participated did not appear normal even when transformed, so this measure was analysed with logistic regression as appropriate for binomial proportion data (see e.g.^27^).

To test whether motivation increased when learning was successful, indicators of motivation were analysed within individuals in the Learning group during the discrimination learning stage. Paired t-tests were used for variables which were normally distributed, and Wilcoxon signed-rank tests where data were non-normal. These tests compared the variable of interest between sessions that contributed to the heifer meeting the learning criterion (i.e. the three sessions or sets of five trials plus the probe session in which they performed at 80% correct or greater, with additional sessions if some were incomplete) and the sessions that prior to these.

To test whether increased play was associated with learning, the mean number of occurrences per session was calculated for each individual. The sample size was 17 for these analyses due to some missing or inadequate videos from the first cohort. Because these data could not meet the assumption of normality, Wilcoxon rank sum tests were used to compare the treatments to determine whether Learning heifers played more than Control heifers once they had the opportunity to learn. Separate tests were run for values during the baseline period to ensure there were no pre-existing differences between treatments. Counts of occurrences of any of the play behaviours were then plotted against learning performance within the discrimination stage for each Learning heifer. These were inspected visually for changes in play corresponding with improvements in performance.

## Supplementary information

Supplementary Information.

## Data Availability

Data used in the analysis are available in the Supplementary Information.
