# Supplementary material for: Assessing the motivation to learn in cattle
Source: Sci Rep. 2020 Apr 22;10:6847. doi: 10.1038/s41598-020-63848-1 (PMC7176709; doi:10.1038/s41598-020-63848-1)
Supplement: Supplementary file 1 — Supplementary Information. [file 41598_2020_63848_MOESM1_ESM.pdf]

Assessing the motivation to learn in cattle

Supplementary material

R.K. Meagher, E. Strazhnik, M.A.G. von Keyserlingk, D.M. Weary

Methods

Training Protocol

All heifers were trained once each day in the morning throughout the procedure. Once some of the cohort reached step 4, they could also voluntarily participate in an additional session two afternoons per week. Beginning at step 2, the trainer sat in the testing area behind a plywood-covered gate, to remain out of sight of the heifers as much as possible, with one small window to allow observation of the operant response.

Step 1: Habituation

Heifers were allowed into the experimental alley for 15 minutes with half of the conspecifics in their pen. A bin containing grain (Hi-Pro Feeds mix described in main text) was placed in the alley in front of the gate to the testing arena. This was repeated for individuals that failed to approach the test area during clicker training sessions (step 2).

Step 2: Clicker training

Heifers were let into the alley in pairs. The bin containing a grain reward was placed in the training area. Latency to approach this area (within 102 cm, based on the position of a wooden post in the wall of the alley, oriented towards the bin) was recorded. When the heifer first approached the bin, the trainer used a clicker (Clik-R, Radio Systems PetSafe Europe Ltd., Dundalk, Co., Ireland) to signal a reward would be delivered; a small handful of grain was delivered into the bin via a tube, such that the trainer's hand was not visible to the animals. The next signal and reward would be delivered when the individual raised her head after eating the grain reward. This procedure was repeated 10 times or until the individual lost interest in the session (as determined by a latency to re-approach of >60 s). Once a heifer received at least 20 pairings of grain and clicker, and eaten at least five for two consecutive sessions, she graduated to the next training step. Heifers that did not meet this criterion within 25 sessions were excluded from the experiment.

The following limits were set for non-participation, due to both practical and welfare considerations. Heifers were initially given a maximum of 6 minutes to approach the training area. If they had approached and performed a response but then ceased to do so, they were given up to

three minutes to return to the task (two minutes if they went to the middle of the alley and one if the far end, due to the decreasing likelihood of returning).

Step 3: Operant response

The operant target (blue plastic lid) was suspended from a rope at the eye level of the heifers, directly above the reward bin and in front of the gate window to be in sight of the trainer. Once the heifer touched the lid with her muzzle, the trainer gave a click then delivered a grain reward as in clicker training. A session was considered complete when the animal received at least nine out of ten possible rewards. After complete sessions on two consecutive days, the individuals progressed to the next stage.

Step 4: Variable interval (VI) schedule introduction

Training was then shifted to a VI schedule such that some operant responses would be unrewarded and the number of responses would differ between individuals. Heifers were rewarded for the first touch of the lid, after which there was an interval during which no rewards were available. This interval was determined by a set of four randomly generated numbers, initially averaging 20 seconds (±1 s) and increasing to 25 and then 30 (VI-30; ± 10 s) over the following sessions. The first touch after this period was rewarded. Beginning at this step, the number of rewards provided in a complete session was five. Animals progressed to the next stage of training when a session was completed on a VI-30 schedule with at least one unrewarded response being performed.

Step 5: Change in reward location

Over a minimum of six days of training, the grain bin was progressively moved away from the lid and into the area behind the gate where the learning task would take place. The bin was not moved to the next location until the heifer had completed a set number of sessions (2 with the bin halfway along the gate, 1 at the far end where it opened, 2 with the gate actually open and 1 where the heifer had to step inside the gate) and consumed at least four of the five rewards offered.

Step 6: Forced choice

The bin lid was progressively introduced until it covered the bin fully and had to be pushed off by the heifer. On some trials, straw was then given in place of grain. Learning heifers always received a grain reward under a lid covered by a red foam sheet and straw under a plain white plastic lid, whereas the control group received rewards with a randomly generated pattern of red and white

lids. For both groups, the location in which the bin was placed varied between left and right following a pre-set pseudo-random pattern, so that side was not associated with the bin contents. A second researcher remained out of sight behind the discrimination testing area to reset the bins between trials. After the feed was revealed and the heifer was given up to 2 min to consume it, she was returned to the other side of the gate. The variable interval began at the time the gate closed behind her.

Step 7: Discrimination learning

Learning heifers were presented with two bins in each trial, one containing grain under the red foam lid and the other, straw covered by the white plastic lid. Heifers could therefore learn to discriminate based on colour and/or texture, with the red foam being the 'correct' choice. The sides on which bins were placed varied in a pseudo-random pattern, with each arrangement occurring at least once during each training session. Once the heifer 'chose' by pushing off a lid, the other bin was removed. The proportion of choices correct was calculated for each session; where sessions were incomplete (<5 trials), they were pooled to calculate proportions over the last three sets of five responses, even if the set was completed over multiple days. Once a Learning heifer had three complete sets of five choices at 80% correct, she was given a probe session with empty bins as described in the main text. After a successful probe trial (≥80% correct), it was considered that the individual had learned the discrimination task.

Control heifers were given only one bin containing the sequence of rewards that her yoked learning partner chose in the corresponding session in step 7. Control heifers received a probe for the corresponding learning heifer probe session, where lid colour was randomized and the reward her yoked partner chose was placed in the bin after she removed the lid.

Analysis: R codes

Data transformation:

```
Learningsummary$logLat7 <- log(Learningsummary$LatApp7 + 0.001, base = exp(1))
```

For alternate analyses with dropped pair:

```
Learningsummary_nomeds <- (Learningsummary[Learningsummary$Pair != 16.2, ])
```

General linear model for motivation variables where assumption of normality met:

```
m.loglatvstmt <- lm(Learningsummary$logLat7 ~ Learningsummary$cohort +
Learningsummary$LatApp4.5 + Learningsummary$Tmt)
summary(m.loglatvstmt)
lat.resid = resid(m.loglatvstmt)
hist(lat.resid)
shapiro.test(lat.resid)
lsmeans(m.loglatvstmt, ~ Tmt)
```

Logistic model used for proportion data:

```
m.volvstmt.logistic <- glm(cbind(Learningsummary$VolDone7,
Learningsummary$VolNotDone7) ~ Learningsummary$cohort +
Learningsummary$VolProp4.5 + Learningsummary$Tmt, family=binomial)
summary(m.volvstmt.logistic)
#gives CIs based on log likelihood calculations
confint(m.volvstmt.logistic)
exp(coef(m.volvstmt.logistic))
exp(confint(m.volvstmt.logistic))
```

#paired wilcoxon signed rank on change in latency

```
wilcox.test (Learningpaired$latChange_C, Learningpaired$latChange_L,Paired=TRUE,
exact=FALSE)
```

Results and Discussion

Play

The patterns of play behaviour over sessions with the discrimination learning phase (Step 7) have been overlaid on the learning success, indicated by proportion of choices that were correct for each of the heifers in the learning treatment in Supp. Fig. 1. Visually, peaks in play often seem to follow sessions with high learning performance, but the behaviour was very variable. Play appeared to be more frequently expressed by the individuals who took the largest number of sessions to learn, perhaps indicating that they were more distracted from the task.

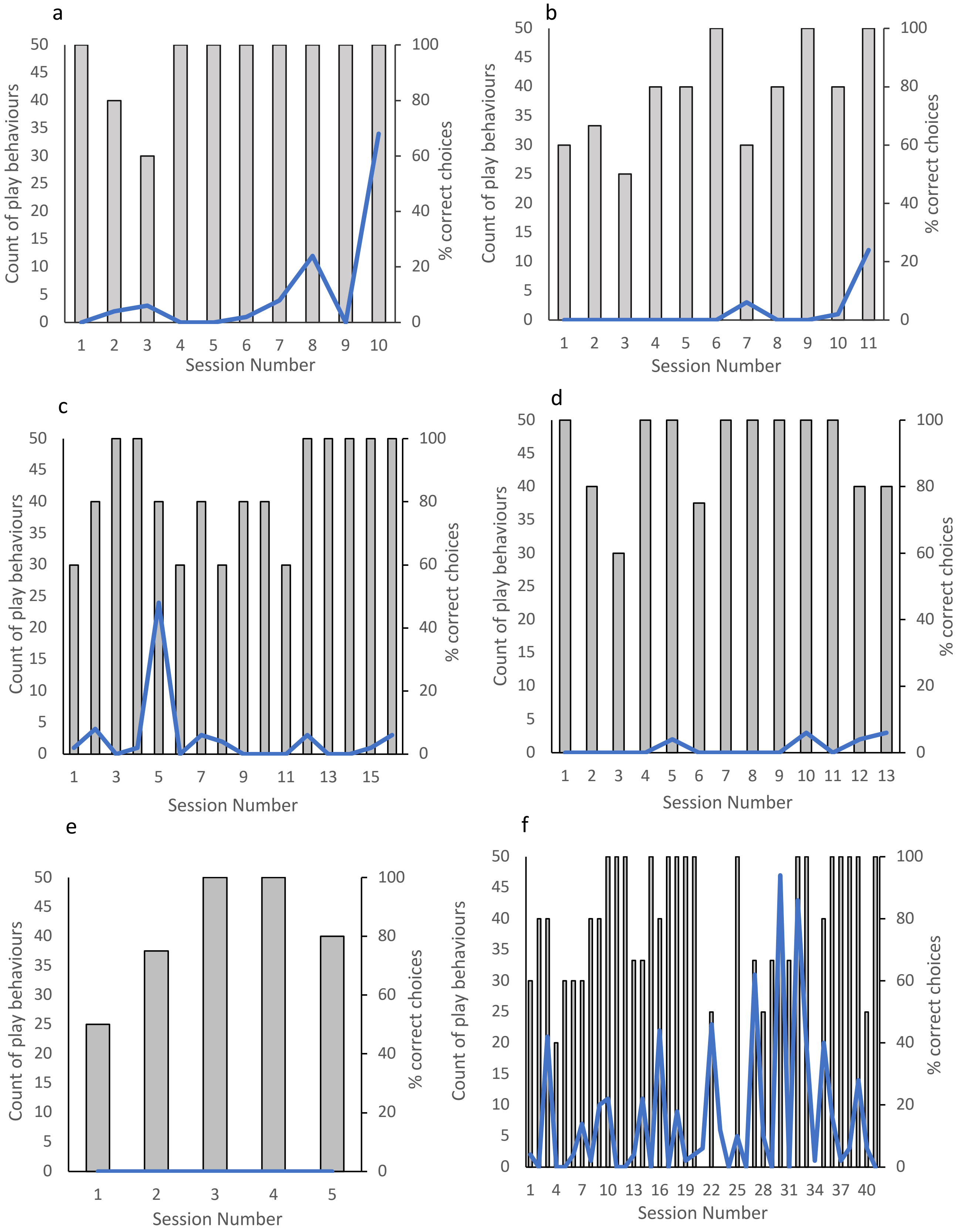
a
Count of play behaviours
% correct choices
50 45 40 35 30 25 20 15 10 5 0
100 80 60 40 20 0
1 2 3 4 5 6 7 8 9 10
Session Number
b
Count of play behaviours
% correct choices
50 45 40 35 30 25 20 15 10 5 0
100 80 60 40 20 0
1 2 3 4 5 6 7 8 9 10 11
Session Number
c
Count of play behaviours
% correct choices
50 45 40 35 30 25 20 15 10 5 0
100 80 60 40 20 0
1 3 5 7 9 11 13 15
Session Number
d
Count of play behaviours
% correct choices
50 45 40 35 30 25 20 15 10 5 0
100 80 60 40 20 0
1 2 3 4 5 6 7 8 9 10 11 12 13
Session Number
e
Count of play behaviours
% correct choices
50 45 40 35 30 25 20 15 10 5 0
100 80 60 40 20 0
1 2 3 4 5
Session Number
f
Count of play behaviours
% correct choices
50 45 40 35 30 25 20 15 10 5 0
100 80 60 40 20 0
1 4 7 10 13 16 19 22 25 28 31 34 37 40
Session Number

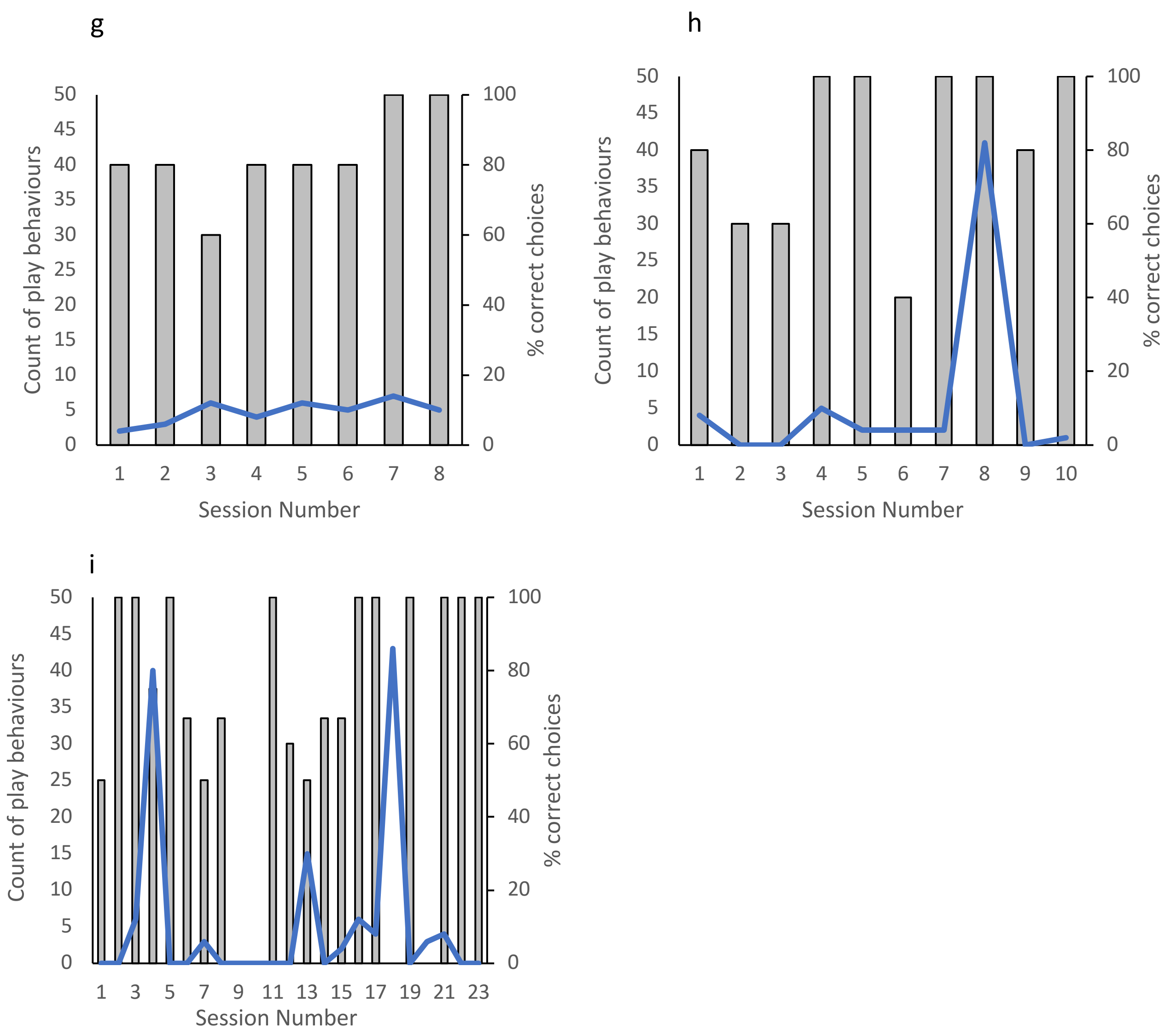

Fig. 1. Play and learning success of Learning heifers by session during discrimination learning. Each chart reflects a single heifer. Bars indicate correct choices in the learning task within a session (whether or not 10 responses performed in that session); line indicates occurrences of any play behaviour.

ID	Tmt	Pair	Cohort	Pen	LatApp4&5	LatApp7	SumInteractions4&5	SumInteractions7	Sessions 4&5	Sessions7	Int/sess 4&5	Int/sess 7	VolProp4&5	VolProp7	VolDone45
4087	C	3	1	2	44.3889	33.8	131	177	15	10	8.733333	17.7	0.5	0.4	1
4092	C	2	1	2	6.05	20.92857	63	134	9	14	7	9.571429	0.5	0.4	1
4094	C	1	1	2	17.4714	157.5	93	71	12	16	7.75	4.4375	1	0.333333	1
4086	L	1	1	2	9.5	10.6875	181	164	16	16	11.3125	10.25		0.25	0
4093	L	2	1	2	3.875	15	71	253	10	14	7.1	18.07143	0.25	0.5	1
4095	L	6	1	2	13.8667	10.09091	79	256	12	11	6.583333	23.27273	0.5	0.4	1
4098	L	3	1	2	9.28571	4	106	115	11	10	9.636364	11.5	1	1	1
4097	C	4	1	3	110.357	59.16667	85	64	14	6	6.071429	10.66667	0.5	1	1
4121	C	6	1	3	11.4286	9.818182	74	38	11	11	6.727273	3.454545	0.75	1	2
5010	C	5	1	3	9.6375	16.5	98	298	13	14	7.538462	21.28571	1	0.5	1
5002	L	4	1	3	23.5222	6.5	107	68	14	6	7.642857	11.33333	1	0.666667	1
5006	L	5	1	3	5.52083	6.846154	90	259	11	14	8.181818	18.5	1	0.833333	1
5090	C	16.1	2	4	52.7323	77.04651	205	231	20	43	10.25	5.372093	0.333333	0.75	2
5094	C	16.4	2	4	46.4683	107.7	122	45	16	10	7.625	4.5	0	0.6	0
5095	L	16.1	2	4	55.95	86.43902	124	185	18	41	6.888889	4.512195	0.5	1	1
5098	C	16.3	2	4	36.9286	153.2083	173	71	14	24	12.35714	2.958333	0.5	1	2
5100	L	16.4	2	4	34.9167	81.8	141	121	12	10	11.75	12.1	0	0.166667	0
5102	L	16.3	2	4	11.8667	117.1304	118	115	11	24	10.72727	4.791667	0.5	0.875	2
5104	L	16.2	2	4	13.05	5.875	99	99	11	8	9	12.375	0.75	1	2
5106	C	16.2	2	4	27.1875	164.6	110	31	12	10	9.166667	3.1	0.25	0	1
